# Modulation of the attentional response to baby schema by testosterone in pedohebephilic men and its relation to the nurturing system

**DOI:** 10.1038/s41598-024-65572-6

**Published:** 2024-07-16

**Authors:** Ronja Zannoni, Julian Keil, Jorge Ponseti, Aglaja V. Stirn, Sarah K. C. Holtfrerich, Esther K. Diekhof

**Affiliations:** 1 Centre for Integrative Psychiatry, Institute for Sexual Medicine and Forensic Psychiatry and Psychotherapy, UKSH, 24105 Kiel, Germany; 2 Department of Psychology, Christian-Albrechts-University Kiel, 24118 Kiel, Germany; 3 Neuroendocrinology and Human Biology Unit, Institute of Cell and Systems Biology of Animals, University Hamburg, 20146 Hamburg, Germany

**Keywords:** Sexual behaviour, Endocrine system and metabolic diseases, Psychology

## Abstract

Previous fMRI research found increased brain responses in men with pedophilic interest to non-sexual pictures of child and animal faces. This raised the question of whether an aberrant nurturing system could be linked to pedophilia. To further explore this hypothesis, 20 pedohebephilic and 23 teleiophilic men performed a target detection task with adult versus infant human and animal faces, which measured selective attention towards the baby schema by comparing reaction times to infant versus adult targets that were presented amongst distractors of the other category. Since the response to baby schema can be influenced by steroid hormones, saliva samples were additionally collected to determine endogenous testosterone, progesterone, estradiol and cortisol. Contrary to expectations, all men did not react faster to infant than adult faces. Yet, pedohebephilic men were more distracted by infant’s faces than teleiophilic men. Pedohebephilic men with higher testosterone were faster in orienting attention to infant targets in the context of adult distractors. This association was not observed in teleiophilic men. Our results support the idea of an overactive nurturing system in pedophilia, which may be influenced by the endogenous testosterone level.

## Introduction

Approximately 1-5% of the male population experience a sexual preference for prepubertal children, which is called pedophilia^1,2^. If the sexual preference is aimed at (early) pubertal children as well as prepubertal children, this is referred to as pedohebephilia^1^. So far, neuropsychological research on the etiology of pedophilia has focused mainly on brain abnormalities related to sexual responses and impulse control^3,4^. However, these results are very heterogeneous, and the etiology of pedophilia remains still unknown^4^. Recent fMRI and EEG studies show that pedophilic subjects had increased brain processing in response to pictures of child faces compared to adult faces, although these were not rated to be sexually arousing^5,6^. In addition, an fMRI study of pedophilic and teleiophilic subjects found a significantly stronger neural response in pedophilic subjects when viewing young animals compared to adult animals^7^. The authors suggest that the preference-specific brain response of men with a pedophilic preference does not depend on the presentation of infant genitals or an infant silhouette as examined in previous studies^8–10^. Based on these findings of an enhanced reaction to infantile non-sexual stimuli in men with a pedophilic preference, the authors raise the nurturing system hypothesis of Eibl-Eibesfeldt^11^, that pedophilia is an eroticization of parental love, which is characterized by nurturing behavior towards offspring and is found both in animals and humans. This thesis assumes that there are organic brain networks that regulate caring and nourishment of offspring (nurturing system) and areas that regulate reproduction (sexual system). These networks have similar physiological functions and use similar neuropeptides^12^. Because the male nurturing system is evolutionarily younger than the female nurturing system, in some men the functional division between a sexual domain and a nurturing domain might be less reliable. Accordingly, in pedophilia, there could be a functionally disturbed separation between these two systems. Following this hypothesis, the aforementioned findings lead to the question, of whether increased brain responses to infant stimuli in pedophilia are rather a consequence of an over-active nurturing system than of an over-active sexual system^7^. Our study is intended to provide further findings to examine this question in subsequent research but does not provide a direct comparison between the two systems.

One known mechanism, that automatically elicits attention and motivates action such as caretaking, is the baby schema effect^13–15^. The baby schema is defined as a set of infantile physical features such as chubby cheeks, a large forehead, and huge eyes^13,16^. Several studies show that the baby schema is a universal stimulus, i.e., it occurs in almost all mammals and functions across species, which is why we find kittens and puppies so cute, for example^13,16–19^. The assistance of alloparents could thus act as a benefit for reproductive success^16,20^. Consequently, the response to baby schema results in increased caring behavior, so that "caring acts" are more likely to be exhibited, such as increased selective attention towards infants. On a neurofunctional level, it activates the oxytocin binding and reward system, in particular the nucleus accumbens^21,22^. Concentration changes of steroid hormones such as testosterone, progesterone, and estradiol as well as cortisol are also considered correlates for experience and behavior in the context of the baby schema^16,23–26^. Several studies suggest a sex difference in the intensity of the reaction to the baby schema^27^. Cárdenas et al.^20^ for example, showed that men were only interested in infant faces when these were presented alongside a male adult face and not with a female adult face. Although testosterone has been largely negatively correlated with paternal behavior, there is some recent evidence that testosterone may play an important role in infant-elicited neural activity and nurturing behavior in men^28^. Nevertheless, the role of hormones in the modulation of nurturing behavior has been hardly assessed, especially in men. To our knowledge, no empirical study has addressed the association between hormones and the response to the baby schema in the context of pedophilia.

The overall research project will explore psychological, behavioral, hormonal, and neurobiological factors that may be important in the development of pedophilia (preregistration on osf.io/3z7pa). The present study used the target detection task (TD) to determine reaction times (RTs) to infants as opposed to adult faces and analyzed the salivary concentrations of testosterone, progesterone, estradiol, and cortisol. By this, we intended to examine whether pedophilic men differ from a male non-pedophilic (e.g., teleiophilic) control group for selective attention to the cross-species baby schema. Building on previous research using the TD paradigm^16,19^ as well as the aforementioned theoretical preliminary considerations of Ponseti et al.^7^, we assumed that the RTs to infantile stimuli are shorter in comparison to adult stimuli of the same species (H_1_), whereby we generally expected shorter RTs to human stimuli in comparison to animal stimuli over both age conditions (H_2_) because of the same-species effect^16,18^. Moreover, we predicted that pedophilic participants react faster to infantile stimuli of all species than teleiophilic participants due to the hypothesis of an overactive nurturing system in pedophilia, which should also be reflected in a faster orienting reaction towards baby schema cues (H_3_)^7^. Based on previous research on the role of salivary testosterone in prosocial behaviors^16,29,30^, we lastly predicted that the endogenous testosterone level should negatively correlate with the speed of attentional orienting to infant pictures in general (H_4_).

## Results

### Behavioral data

The ANOVA revealed a significant main effect of target [*F*(1.00, 40.00) = 4.18, *p *= 0.048, η_p_^2 ^= 0.095], rating [*F*(1.63, 65.18) = 164.11, *p *< 0.001, η_p_^2 ^= 0.804], and a significant interaction between target and rating [*F*(1.72, 68.70) = 9.56, *p *= 0.001, η_p_^2 ^= 0.193]. There were no significant differences in ratings of sexual arousal, valence, and unspecific arousal between pedohebephilic and teleiophilic participants for all conditions. Both groups did not perceive the human pictures as sexually arousing as indicated by the mean ratings (Table S1 [supplementary material]).

### Absolute RTs

Averaged RTs (in ms) of the adult and infant human faces are shown in Table 1. We performed an ANOVA with target (adult vs. infant) as a within-subject factor and group (pedohebephile vs. teleiophile) as a between-subjects factor. To exploratively control for potential confounding variables, the sexual orientation of the participants, pet ownership, and having children were eliminated from the model (neither main effects nor interaction effects were found for these, all *F* < 0.51, all *p* > 0.356). A significant main effect of target [*F*(1, 40) = 7.48, *p* = 0.009, η_p_^2^ = 0.157] and a significant interaction effect between target and group [*F*(1, 40) = 5.09, *p* = 0.030, η_p_^2^ = 0.113] were found (Table S2).

**Table 1:** Averaged RTs for the human image category, given by pedohebephilic and teleiophilic participants [in ms].

	Human adult	Human infant
Pedohebephilic men (*n* = 20), *M* (SD)	1309.10 (149.56)	1317.03 (173.92)
Teleiophilic men (*n* = 22), *M* (SD)	1207.10 (130.76)	1289.89 (140.94)

**Figure 1:**
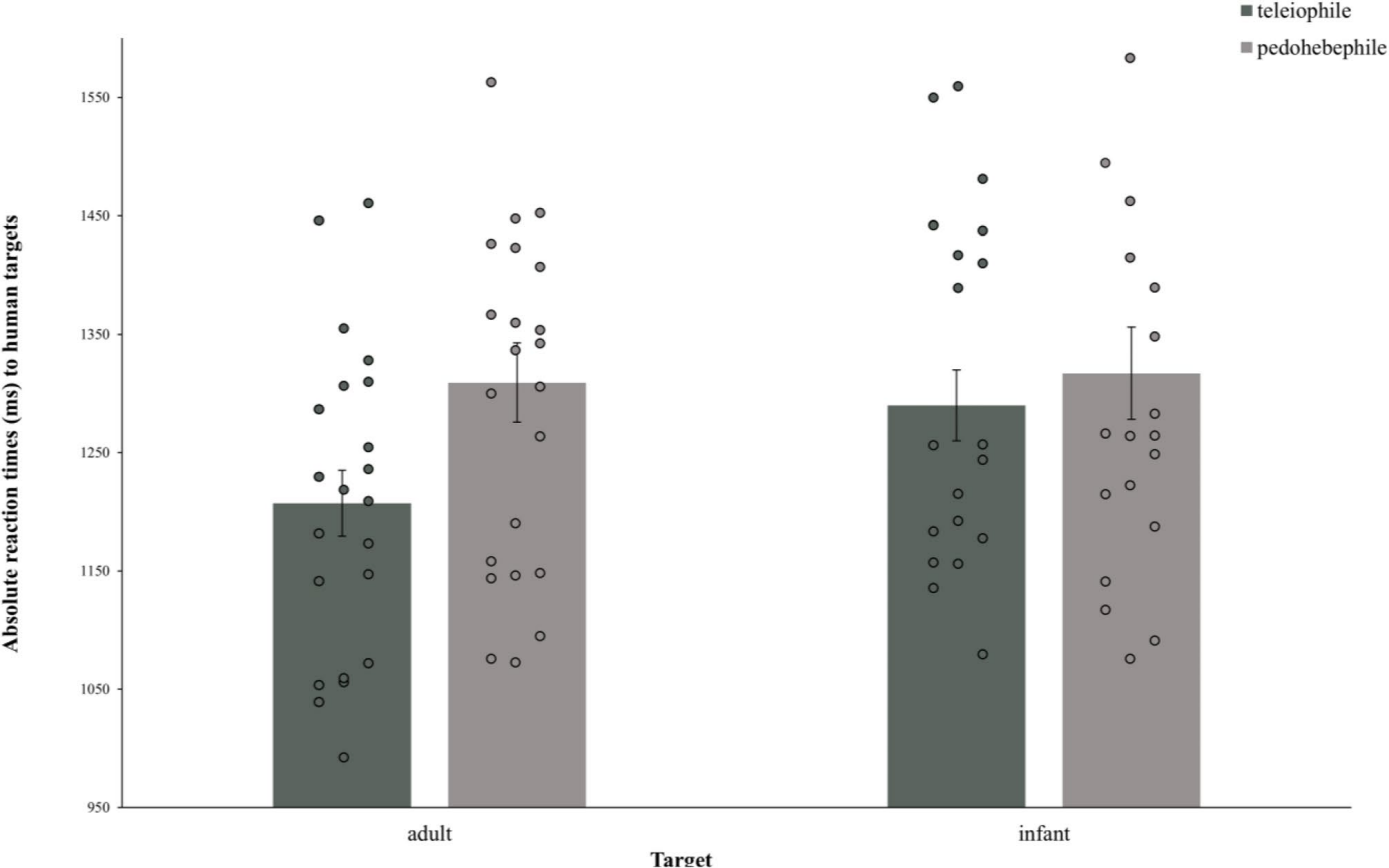
Mean absolute RTs to human targets separated by groups (error bars indicating ± SEM; dots indicating mean absolute RTs per participant).

In particular, Bonferroni-adjusted post-hoc *t*-tests revealed significantly longer RTs for the infant than for the adult human images only in teleiophilic men but not in the pedohebephilic group (*t*(21) = − 3.52, *p *= 0.002, *d *= − 0.75, two-sided) (Figure 1). Further, pedohepephilic participants needed more time to detect the adult human images than the teleiophilic participants (*t*(40) = − 2.36, *p *= 0.023, *d *= − 0.73, two-sided). The interaction effect was still significant when controlling for all hormone levels separately as covariates (all *p *< 0.046).

### Hormone levels

The mean testosterone concentration of the participants was *M* = 139.29 ± *SD *= 51.66 pg/mL (Table 2). Testosterone concentration did not differ between pedohebephilic and teleiophilic participants (*M*_*pedohebephilicgroup*_ = 128.70 pg/mL [*SD* 54.49]; *M*_*teleiophilicgroup *_= 148.92 pg/mL [*SD* 48.15]; *t*(40) = 1.28, *p *= 0.209). There were also no significant differences between both groups for progesterone (*M*_*pedohebephilic *_= 37.85 pg/mL [*SD *= 15.60], *M*_*teleiophilic *_= 45.08 pg/mL [*SD *= 25.36]; *t*(40) = 1.09, *p *= 0.281), estradiol (*M*_*pedohebephilic *_= 3.92 pg/mL [*SD* = 1.73], *M*_*teleiophilic *_= 4.21 pg/mL [*SD *= 2.98]; *t*(40) = 0.379, *p *= 0.707 and cortisol (*M*_*pedohebephilic *_= 9.93 ng/mL [*SD *= 4.93], *M*_*teleiophilic *_= 10.31 ng/mL [*SD *= 4.11]; *t*(40) = 0.279, *p *= 0.782).

**Table 2:** Averaged salivary hormone concentrations separated by groups and across all subjects [testosterone, progesterone, and estradiol in pg/mL; cortisol in ng/mL].

	Testosterone	Progesterone	Estradiol	Cortisol
Pedohebephilic men (*n* = 20), *M* (*SD*)	128.70 (54.49)	37.85 (15.60)	3.92 (1.73)	9.93 (4.93)
Teleiophilic men (*n* = 22), *M* (*SD*)	148.92 (48.15)	45.08 (25.36)	4.21 (2.98)	10.31 (4.11)
Overall (*n* = 42), *M* (*SD*)	139.29 (51.66)	41.64 (21.48)	4.07 (2.44)	10.13 (4.46)

### Hormone levels and absolute RTs

**Figure 2:**
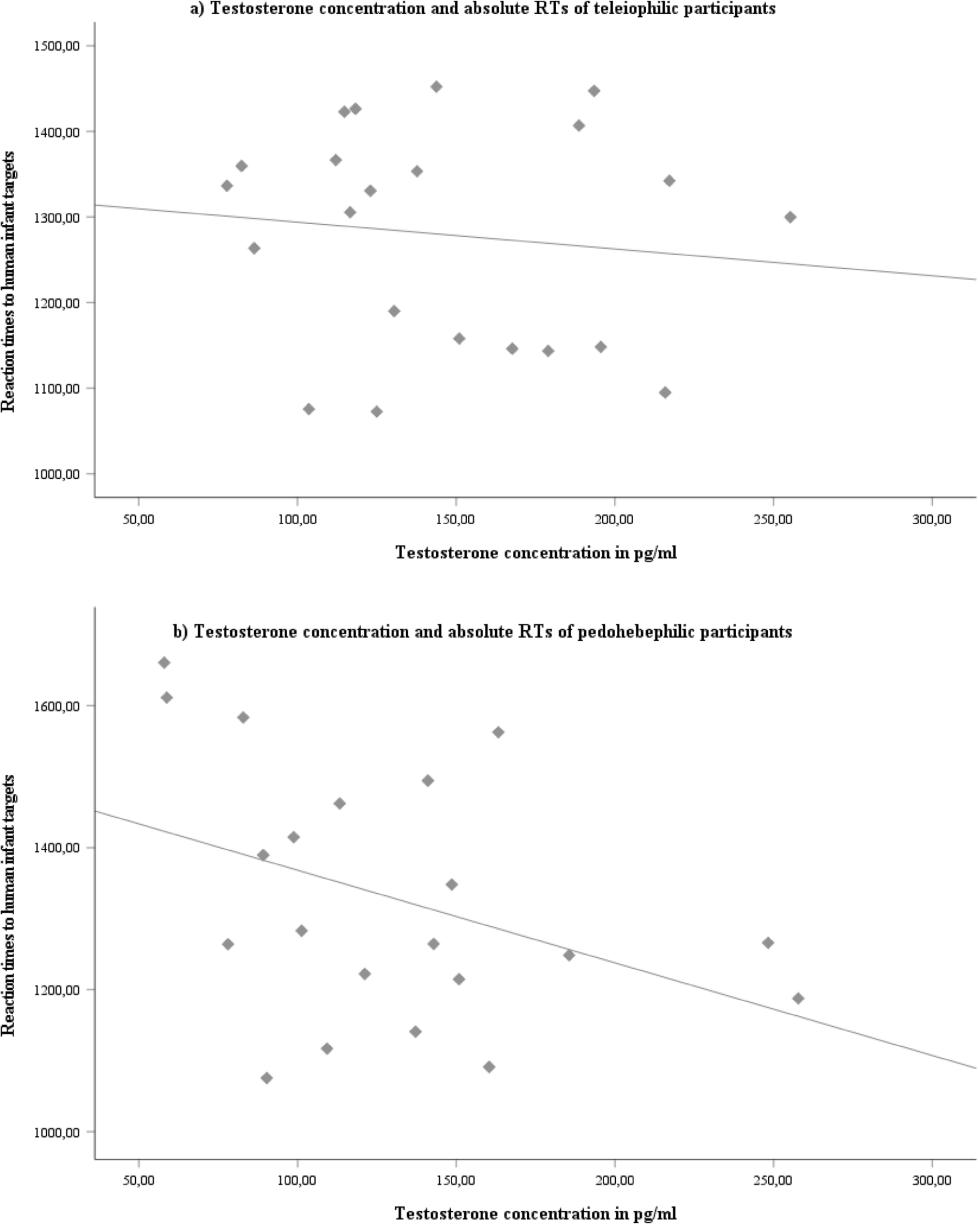
(**a**) No significant negative correlation between salivary testosterone concentration and absolute RTs [in ms] towards human infant portraits in the teleiophilic group (*r* = − 0.070, *p* = 0.757, two-sided). (**b**) Significant negative correlation between salivary testosterone concentration and RTs [in ms] towards human infant portraits in the pedohebephilic group (*r* = − 0.462, *p* = 0.040, two-sided).

When considering the complete sample, there were no significant correlations between the respective salivary hormone level and the RTs for both adult and infant human images (e.g., for testosterone *r*_*adult *_= − 0.276, *p *= 0.076; *r*_*infant *_= − 0.299, *p *= 0.054, two-sided). When looking separately at the two groups, there was a significant negative correlation between testosterone and the RTs to infant human stimuli only in the pedohebephilic group (*r *= − 0.462, *p *= 0.040, two-sided) (Figure 2). However, the comparison of the correlation coefficients between both groups was not significant (*z *= 1.307, *p *= 0.096, one-sided). The other hormones were not correlated with RTs in either of the two groups (all *p *> 0.239).

### Relative RTs (*Delta*-RTs)

**Figure 3:**
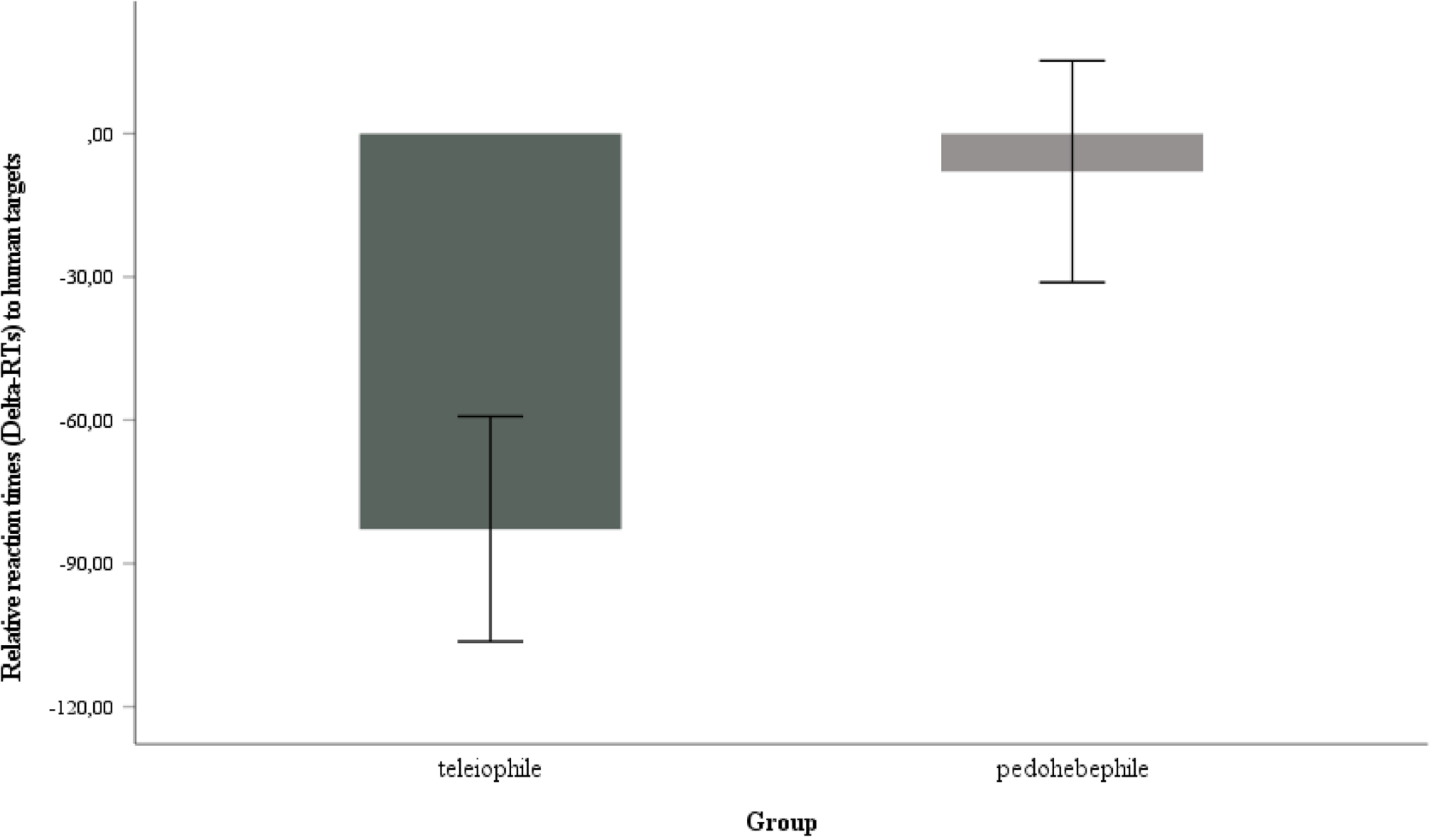
Mean relative RTs (Delta-RTs) to human targets separated by groups (error bars indicating ± SEM).

To explore the Delta-RTs (adult target—infant target) as an index of selective attention for infant targets, we performed an ANOVA on the Delta-RTs of the human targets with *group* (pedohebephile vs. teleiophile) as a between-subjects factor, and the respective *salivary hormone levels* (testosterone, progesterone, estradiol, and cortisol) as covariates in separate ANOVAs. When controlling for all hormones separately, we always found a significant main effect of group (e.g., for the ANOVA that included testosterone as covariate: [*F*(1, 39) = 5.58, *p* = 0.023, η_p_^2^ = 0.125]). Bonferroni-adjusted post-hoc *t*-tests revealed higher Delta-RTs for human images in the pedohebephilic group than in the teleiophilic group (*M*_*pedohebephilic*_ = − 7.92 [*SE* = 23.23], *M*_*teleiophilic*_ = − 82.80 [*SE* = 23.55], *p* = 0.030, *d* = − 0.70, two-sided) (Fig. 3).

### Hormone levels and relative RTs

Over all participants, there were no significant correlations between salivary hormone levels and the Delta-RTs for human targets (all *p* > 0.227). There were also no significant correlations between salivary hormone levels and the Delta-RTs for human targets when looking separately at the two groups (all *p* > 0.085).

## Discussion

The current study aimed to contribute to the question, of whether there is evidence for an aberrant nurturing system in men with a sexual preference in children. Using the TD paradigm, we investigated the connection between endogenous hormone levels known to be relevant for nurturing behavior (testosterone, progesterone, estradiol, and cortisol) and selective attention to infant faces in adult pedohebephilic and teleiophilic men. To examine nurturing processes independent of sexual processes, we showed subjects images of infant and adult male faces, as well as faces of infant and adult cats and dogs, which were rated as sexually non-arousing by all subjects. Unfortunately, due to high error rates in the animal conditions, only RTs to human stimuli could be analyzed. Thus, we could not examine cross-species effects (esp. H_2_).

In contrast to our first hypothesis that RTs to infant stimuli would be shorter than to adult stimuli, teleiophilic participants needed more time to detect infant than adult human targets. There was no significant difference in RTs to adult and infantile faces in the pedohebephilic group. Interestingly, pedohebephilic subjects needed more time to detect adult targets than teleiophilic subjects. However, they did not react faster to infantile stimuli than the teleiophilic subjects (H_3_). The results contradict the two hypotheses and the studies by Holtfrerich et al.^16,19^, however, this is the first TD study with male pedohebephilic and teleiophilic subjects, and previous results predominantly referred to young, nulliparous women. Although the baby schema-specific neural response has been observed in both women and men, there is evidence of a sex difference in this response^31^. Both ERP studies and behavioral measures showed that the baby schema effect was more pronounced in women than in men^32–34^. This sex difference has been shown when infant faces were provided as viewing options along with same-sex and opposite-sex adult faces^32,33^ as well as when animal faces were used as additional viewing options^35^. Interestingly, our results fit with those of previous studies demonstrating an own-age bias in face detection^36–38^. Using a breaking continuous flash suppression (bCFS) with infant and adult faces, Stein et al.^38^ found that adult faces reached awareness more quickly than infant faces. An explanation for this effect could be, that a perceiver’s experience with members of their own social group facilitates access of those faces to awareness^37,39^. These phenomena are also referred to as own-group (own-ethnicity, own-gender, and own-age) biases and are revealed through faster and more accurate recognition of faces of one’s own-group relative to those of out-groups^40,41^, which can be explained in terms of differences in interest and motivation^42,43^ or perceptual expertise^44^. Since we did not explicitly examine own-group biases, we can only speculate about the influence of individual biases. Our stimulus set consisted of babies, in whom gender was not identifiable, and middle-aged males, all Caucasian, which means the adult stimuli were comparable to our male sample. Accordingly, since only the age of the faces shown visibly varied, an own-age bias may have caused the RT advantage in adult faces. This hypothesis is consistent with a previous visual search task in which the search efficiency was greater for adult faces than for infant or child faces in adults with limited experience with infants or children but not in subjects with extensive recent experience with children^36^. However, in our present study, parenting had no significant effect on RTs. Assuming that especially teleiophilic men are more familiar with male faces of the same age than with infant faces and considering our conservative stimuli selection of both adult and infant faces with low baby schema proportion, an own-age bias could have worked against the baby schema effect. This would fit in particular with the result that only the teleiophilic subjects took significantly longer for the infant faces but not the pedohebephilic group.

Interestingly, pedohebephilic subjects took significantly longer to identify adult stimuli than teleiophilic subjects. Moreover, they were significantly more distracted by the infantile stimuli, when these surrounded an adult target, than the teleiophilic group. This effect was not found for infant targets with adult distractors. Although we can only speculate due to some relevant limitations (including our heterogenous sample composition and size), the significantly higher Delta-RTs for the pedohebephilic group could thus represent the distractor effect but not the target effect of the infants. An explanation for this group difference could be the salience of infant cues for pedohebephilic men due to their sexual preference. Their attentional bias towards the infantile distractors could be caused by the fact that they might be more involved in infant cues in their daily life than teleiophilic men and that these stimuli are therefore of greater salience. However, this interaction effect can only be interpreted with limitations since it only occurred in the final group assignment. Possibly, subject-specific factors could also have an impact.

Salivary hormone levels were analyzed to test for correlations between the endogenous hormone status and men’s selective attention toward infant pictures. Pedohebephilic and teleiophilic subjects did not differ significantly in the concentration of testosterone, progesterone, estradiol, and cortisol. This is in line with recent studies showing, contrary to the general belief, that individuals with a sexual preference for children (both child sexual offenders and non-offenders) do not have altered testosterone levels compared to teleiophilic subjects^45^. However, because there are currently no comparative studies with non-offending pedophilic subjects, further research is required to determine if and how these hormones and particularly testosterone play a role in pedophilia. Further, we found a negative correlation between the endogenous testosterone level and the absolute RTs for infant targets only in pedohebephile subjects, meaning that the higher their testosterone level was, the faster they were able to detect infant faces amongst adult distractors. However, the correlation of the pedohebephilic group was not statistically significantly greater than the correlation of the teleiophilic group. For the other hormones, there were no significant correlations with absolute and relative RTs in both groups.

Sex hormones such as testosterone, estradiol, progesterone, and cortisol have long been known to influence mating behavior but also parental responsiveness and interest in infants^25,28,46–48^. To date, they have been studied primarily in mothers and never in the context of nurturing processes in pedophilia. Most studies on hormones and deviant sexual behaviors have been in forensic settings investigating testosterone levels of sexual offenders^49^. Contrary to the general belief, it is nowadays known that men with a sexual preference for children do not have altered testosterone concentrations^50^, which is in line with our results. However, our negative correlation between testosterone and absolute RTs for infant faces in pedohebephile subjects is contrary to Holtfrerich et al.’s^16,19^ finding in young women. Various explanations are conceivable. Sexual attractiveness and cuteness are two main characteristics of facial attractiveness in human faces^51^. Therefore, the facial cuteness of infant faces may affect the attention and other cognitive processing of such faces^52,53^ as well as the reward system^14^. At the behavioral level, an attentional bias towards highly attractive faces has been found to both adult and infant faces, as indicated by reduced RTs or faster eye movement^54–57^. Studies considering the perspective of mate selection have shown that the attentional bias to highly attractive faces is strongly affected by biological factors such as the sex of the targets and the hormone level of the observers. Hahn et al.^32^ revealed that intra-individual variation of the reward value of a baby’s face is dependent on testosterone levels. For our results, infant faces may be highly attractive and/or rewarding for pedohebephilic subjects, although there were no significant differences in subjective ratings of sexual arousal, valence, and unspecific arousal between groups. However, the attractiveness of the baby schema is made up of other determinants such as perceived cuteness^14,17,58^, and it could therefore be that these are particularly salient for pedohebephilic subjects. Additionally, testosterone is known to be associated with both the reward value of infant cues and the sexual salience of stimuli^59,60^. This could possibly have influenced the negative correlation of testosterone and the RTs related to infant cues. Neuroendocrinological studies with adult heterosexual men show that basal testosterone levels are associated with both mating and nurturing behavior^61–63^. However, according to a recent review, results on the relationship between testosterone, mating and nurturing processes are inconsistent and highly dependent on the social context of the cues as well as individual differences in personality and biological traits of the observer^64^. As there are no comparable studies on pedohebephilic subjects and our sample was relatively small, we can only speculate about the negative correlation found between testosterone and RTs to infant cues. An evolutionary psychological hypothesis would be that the trade-off between sexual and caring processes might work differently, such that higher testosterone levels are associated with increased attention to and reward processing of baby schema cues. Thus, care intentions might serve as mating signals for further reproductive goals^65^ instead of being in opposition^58^. However, our experimental TD design is not sufficient to provide empirical evidence for such a hypothesis. Above all, future neuropsychological research, such as fMRI or EEG studies on the trade-off between sexual and nurturing processes, is needed to answer these open questions. Despite that, our study provides preliminary evidence that pedohebephilic subjects are more distracted by non-sexual baby schema cues and that in particular those with higher testosterone levels show increased attention towards the baby schema.

Some limitations of the study should yet be discussed. First, the pedohebephilic group was mostly non-exclusive (*n *= 17) and a clear group assignment was difficult for some subjects, as they were also on the spectrum of hebe- and/or teleiophilia. It cannot be assumed that the same mechanisms play a role in the etiology of pedophilia and hebephilia, nor that infant images have the same effect on exclusive and non-exclusive pedophilic subjects. However, since all subjects in the sample had at least pedophilic proportions (no exclusively hebephilic subjects), we would expect even larger effects on RTs in a more homogenous sample of exclusively pedophilic subjects. Second, both of our groups were relatively small due to the difficulty of reaching people with a sexual preference for children and because we had to conduct the study during the COVID-19 pandemic. This means that even though some contrasts yielded significant results, small changes in participant characteristics can lead to fluctuations in the results^66^. Although difficult to attain, replications with larger samples of men with an exclusive sexual interest in children are needed to corroborate these effects with more certainty. Third, our stimulus material of domesticated animals was inappropriate for a valid analysis, as shown by the high error rates and participant feedback. Almost all subjects reported back that the adult and infantile dogs and cats were almost indistinguishable. This could be because British Shorthair cats per se have a high baby schema and German Shepherds per se have a low baby schema, so the infants and adults were hardly distinguishable. In addition, both breeds have a strong hairiness, which could have made discrimination even more difficult. To investigate cross-species effects of the baby schema in pedophilia, other cat and dog breeds with the same coat color and little hairiness should be chosen as stimuli, e.g., European shorthair cats and Labradors. Fourth, it must be taken into account that for the human faces we used only those with low baby schema, since no normal faces were available in the data set, and we did not want to create artificial effects with the exclusive presentation of high baby schema. This may have resulted in potential effects going undetected or possibly being larger if unmorphed faces had been used. A follow-up study with unmorphed human and animal faces across all conditions would be recommended. Fifth, subjects were asked to assess saliva samples after waking up at home, as this method is non-invasive, and samples are easy to collect. However, hormone levels in saliva can fluctuate from moment to moment and are influenced by factors such as momentary emotional states and food intake^67^. In addition, we cannot retrospectively verify that all subjects collected samples adequately. However, since the samples should have been taken directly after getting up when the steroid hormones approximated their morning peak and saliva was still uncontaminated by food intake or other daily factors like stress, it was difficult to realize a controlled collection in the laboratory. If possible, the analysis of oxytocin and vasopressin via blood sampling would also be of highest interest for future studies, as these cannot be obtained via saliva but play a relevant role in nurturing behavior^16,19,68^. Finally, correlational analyses are unfit to prove causation and can increase the risk of third variable confounds in case-control study designs^66^. Despite these limitations, our findings can be understood as providing substantial guidance for future studies in the area of basic research on potential associations between nurturing processes and sexual preference for children.

This study provides empirical evidence for aberrant visual processing of non-sexual infant stimuli in pedohebephilic men. Pedohebephilic subjects were in general more distracted by the baby schema. Additionally, there is evidence that high endogenous testosterone in pedohebephilic men promotes selective attention in infants. Future studies with larger, homogeneous samples and appropriate, standardized stimuli material of animals are needed to investigate cross-species effects of the reaction towards the baby schema independent of sexual processes.

## Methods

### Participants

Pedohebephilic subjects were recruited from the outpatient clinics of sexual medicine in Kiel and Hamburg, or the community. The survey period was from December 2020 to April 2022. Recruitment from the community was done by re-contacting subjects of a former project on the neurobiological mechanisms underlying pedophilia (www.nemup.de) as well as by informing self-identified pedohebephilic men via various German websites for people with such sexual interests and via our institute website. The study participants with a teleiophilic preference were recruited via postings at universities and other local facilities, e-mail distribution lists, and by announcements via social media. All participants gave written informed consent for every part of the study in which they participated and were compensated for full participation (50€ and, in justified cases, the assumption of travel expenses to the laboratory).

Inclusion criteria for all participants were an age of at least 18 years, exclusively male, no current alcohol or substance abuse as well as no intake of mood- or attention-changing medication at the time of the study, whereas patients with other comorbidities could be included. Originally, more restrictive exclusion criteria were specified (a disorder of sexual preference, for the experimental group a diagnosis or self-reported preference for zoophilia, and for both groups psychological or neuronal disorders), but due to the selective sample, subjects who did not meet all criteria were finally included. Confounding variables potentially relevant to the study design (e.g., self-reported zoophilia) were controlled. Pedohebephilic participants were assigned based on orientation to the formal criteria for pedophilia ICD F65.4, participants of the control group (teleiophilic men) had to be primarily oriented towards the adult body schema (Tanner 4–5^69^) and must not have committed sexual assaults on persons under the age of 18 years. Initially, only subjects with an exclusively pedophilic preference were to be recruited for the experimental group. However, since only 3 out of 20 subjects had exclusive pedophilia, we also included subjects with pedohebephilic tendencies. The final sample (*N *= 43) consisted of 20 pedohebephilic subjects (primary preference for child-like, or juvenile-like body schemes of Tanner 1–3) and 23 teleiophilic subjects (primary preference for adolescent-like, or adult body schemes of Tanner 4–5). Groups were matched for age (*M* [*SD*], pedohebephilic men: 40.30 [15.60] years, teleiophilic men: 34.04 [9.94] years; *t*(31.40) = 1.54, *p *= 0.133, two-sided), IQ as measured by the German short form of the Cattell’s Fluid Intelligence Test, Scale 2 (CFT 20-R; Weiß, 2006) (*M* [*SD*], pedohebephilic men: 109.45 [17.91], teleiophilic men: 115.83 [17.88]; *t*(41) = − 1.17, *p *= 0.251, two-sided), height in cm (*M* [*SD*], pedohebephilic men: 183.60 [6.64], teleiophilic men: 182.09 [4.79]; *t*(41) = 0.87, *p *= 0.392, two-sided), handedness assessed by Edinburgh Handedness Inventory (EHI; Oldfield, 1971) (*M* [*SD*] of the EHI Laterality Index, pedohebephilic men: 66.78 [51.53], teleiophilic men: 86.15 [16.50]; *t*(22.38) = − 1.61, *p *= 0.121, two-sided) and sexual gender orientation (70 % heterosexual pedohebephilic men, 30 % non-heterosexual pedohebephilic men, 91.3 % heterosexual teleiophilic men, 8.7 % non-heterosexual teleiophilic men; χ^2 ^= 3.206, *df *= 1, *p *= 0.073; ϕ = − 0.273, two-sided) (Table S3).

### Measures

The TD was part of an overall project to explore psychological, behavioral, hormonal, and neurobiological factors that could be of importance in the etiology of pedophilia. All subjects completed an online questionnaire in advance and collected saliva samples at home. On the test day in the laboratory, a passive viewing experiment with EEG measurement and the TD were performed, followed by the German short form of the Cattell’s Fluid Intelligence Test, Scale 2 (CFT 20-R^70^) to measure fluid intelligence as well as a modified German form of the Self-Assessment Manikin (SAM^71^) to assess subjects’ ratings of the faces presented in terms of sexual arousal, valence, and unspecific arousal. The results from the EEG measurement are not part of the present study and will be published in a separate manuscript. The study was conducted in accordance with the 2008 Declaration of Helsinki and ethical approval was obtained by the Ethics Committee of the Medical Faculty of the University of Kiel (No. D445/20). All participants gave written informed consent for all parts of the study.

### Sexual interests and inclusion criteria

All participants received a personalized link to an online questionnaire that was conducted via the platform SosciSurvey before the test day. The online questionnaire was based on a semi-structured interview^10^ to collect sociodemographic data, neurological and developmental difficulties, delinquency history, information on sexual age and gender preference for group assignment, and inclusion criteria for study participation. Sexual age and gender preference (as well as the offense history) were assessed via self-report and then confirmed by presenting participants the Growth Diagrams^69^, asking them to rate their age and gender preference in terms of sexual fantasies and actual sexual acts using a modified version of the Kinsey scale for developmental stages^72^. Additionally, a viewing-time paradigm (VT)^73^ was utilized to ensure valid group classification. The online questionnaire also included the German version of the Patient Health Questionnaire (PHQ-D^74^) to screen for psychological disorders as well as the German version of the Edinburgh Handedness Inventory to test for right- or left-handedness^75^.

### Sampling procedure

On the morning of the test day, participants each collected three saliva samples in Eppendorf microcentrifuge tubes (2 ml) 30 min apart after waking and brought them to testing. Before collection, participants were instructed not to eat, smoke, or consume products that could affect hormone measurement during sampling. Subjects were allowed to drink water between collection intervals as well as to brush their teeth directly after the first sample collection (to avoid blood contamination), but only until five minutes before the sample collection. The samples were then frozen at − 20 °C until analysis in the ISD Laboratory (Malente, Germany). There, the three samples were thawed and, centrifuged and equal amounts of the aliquot were pooled for analysis with commercial enzyme-linked immunosorbent assay (ELISA) kits: Salivary Testosterone ELISA DES6622 with an analytical sensitivity of 6.1 pg/mL and inter-and intra-assay coefficients of variation below 9%; Salivary Estradiol ELISA SLV-4188 with a sensitivity of 0.4 pg/mL and inter-and intra-assay coefficients of variation below 7%; Salivary Progesterone ELISA SLV-2931 with a sensitivity of 3.8 pg/mL and inter-and intra-assay coefficients of variation below 8%; Salivary Cortisol ELISA DES6611 with a sensitivity of 0.019 ng/mL and inter-and intra-assay coefficients of variation below 8% and 10%, respectively.

### Visual stimuli of the TD

**Figure 4:**
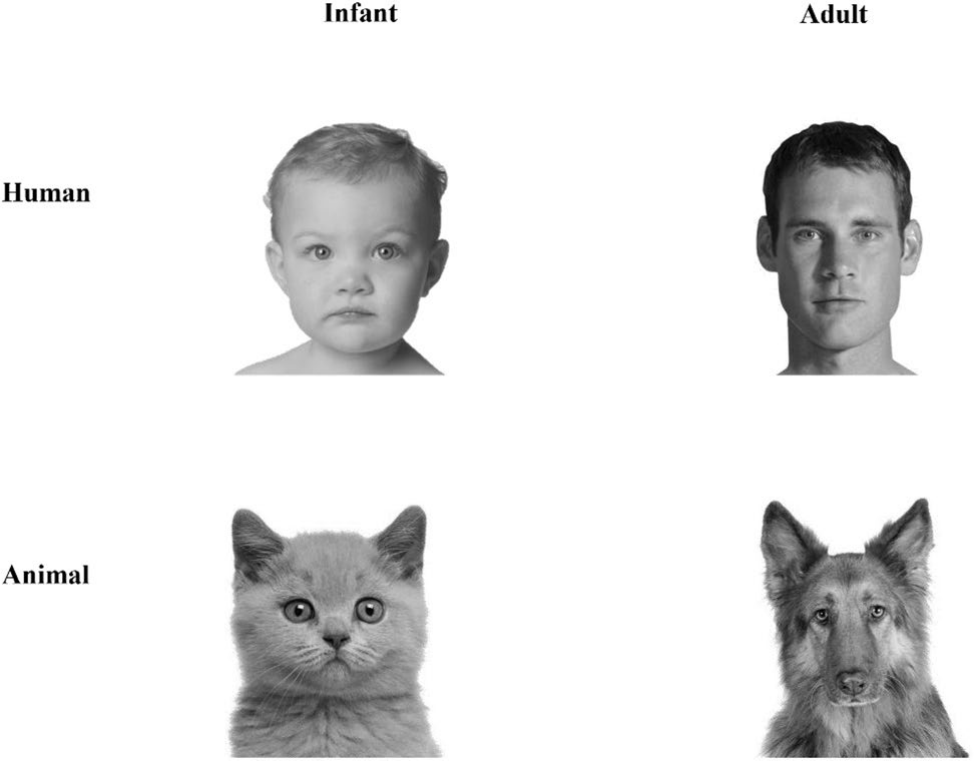
Examples of presented stimuli to participants. Infant faces of a human and a British shorthair cat on the left, adult faces of a male human and a German shepherd on the right. Photos above: Thinkstock/Getty Images (Borgi et al.^17^; modified); photos below: Adobe Stock (https://stock.adobe.com/de/; modified with MATLAB R2020a (https://de.mathworks.com/products/new_products/release2020a.html)).

Four male faces and 4 human baby faces as well as 4 adult and infant faces of dogs and cats were shown. Stimuli depicting standardized human adult and infant faces with neutral facial expressions were provided by Borgi et al.^17^. The pictures were digitized at 72 dpi and were two-dimensionally rotated and scaled to a head length of 600 pixels. Using a coordinate system on the faces, facial landmarks were measured and then parametrically manipulated using Adobe Photoshop to create high (round face, high forehead and large eyes, small nose and mouth) and low (narrow face, low forehead and small eyes, large nose and mouth) baby schema portraits of each subject (following Glocker et al.^14^, for a detailed overview of the stimulus creation procedure, see Borgi et al.^17^). From this data set, we exclusively used 2 adult male faces and 4 infant faces, all with low baby schema. We only used adult male faces to avoid triggering activation of sexual areas in heterosexual controls (originally, only heterosexual male subjects were planned for the control group; however, homosexual subjects eventually participated as well. The influence of sexual orientation on the variables of interest was controlled and had no significant impact [see results section]). For the human infant stimuli, the children depicted were so young that no gender was obvious. To avoid creating artificial effects, we opted for a conservative approach and used only human stimuli with low baby schema (instead of high baby schema, since no standardized, unmanipulated images were available). Following the same procedure as Borgi et al.^17^, we used Adobe Photoshop to parametrically manipulate 2 more adult human males with low baby schema. For the animal stimuli, we used 8 photographs of one cat breed (4 adult and 4 infant British shorthair cats) and 8 photographs of one dog breed (4 adult and 4 infant German shepherds), in each of which no gender was recognizable; all pictures were obtained from Adobe Stock. The two breed types were selected for their uniform coat color and a not-too-strong natural child pattern at adult age. The animal faces were not manipulated regarding the baby schema. All pictures were standardized, converted to greyscale using MATLAB, brought to the same size, and were visually equalized in luminance. The total stimulus set consisted of 24 test images, which were divided into 2 (age: adult vs. infantile) × 3 (species: human vs. cat vs. dog)—conditions, i.e., 4 test images are presented per condition. For an example of the manipulated stimuli see Figure 4.

### Experimental design of the TD task

**Figure 5:**
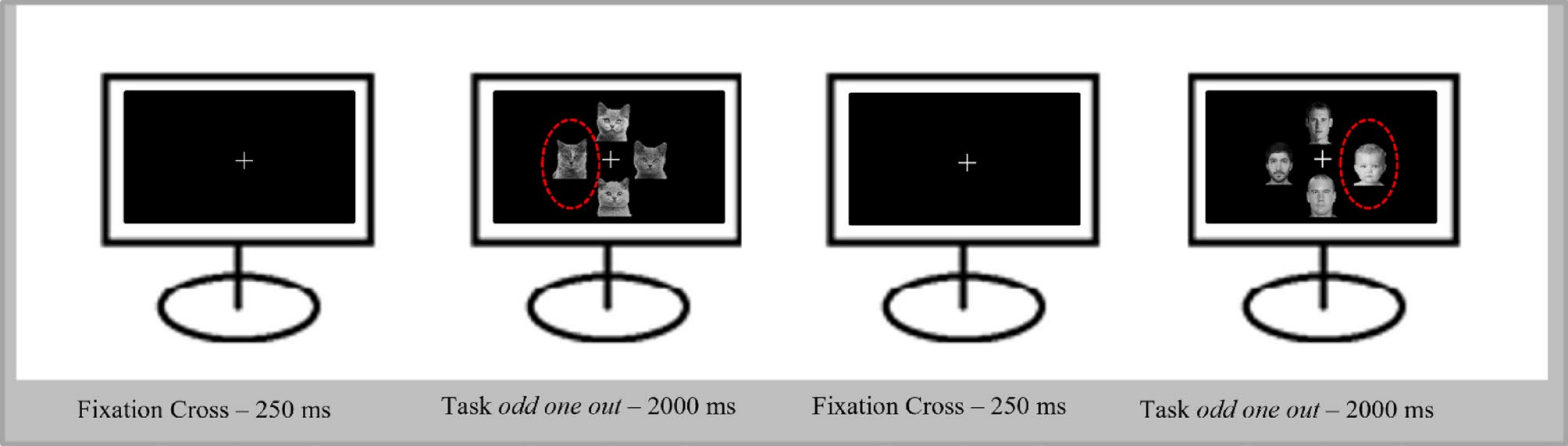
Example illustration of two trials out of the TD paradigm.

The TD was based on the *odd-one-out* principle. The participant was instructed to select one stimulus out of four faces that did not fit in the crowd (i.e., the infant among three adults, or the adult among three infants) via keypress as fast and as accurately as possible. The key positions conformed with the picture positions. During this procedure, the participant’s response time to the targets was measured. The duration between the appearance of the stimulus and the key press was associated with the selective attention to the stimulus attracted^16^. Each trial consisted of four pictures of the same species that were arranged in a cross pattern. In each trial, either three infants (human or animal) and one adult target or three adults in combination with one infant target were shown (see Holtfrerich et al.^16,19^, for a similar procedure). Before the actual experiment, a trial run was conducted with other stimuli (taken from Holtfrerich et al.^16^) to ensure instructional comprehension and correct study performance. The main experiment consisted of 150 trials. The faces were cropped from their original background and presented on a black square sized 319 pixels against a black background. The images were shown 50 times per species, i.e., 25 repetitions per adult and infantile target. Each trial had a duration of 2250 ms (Figure 5). The order of the trials was pseudo-randomized and balanced for trial-type transitions to avoid more than two repetitions of the same condition. If a trial was missed, the stimulation continued after 2000 ms automatically. The whole paradigm was performed in a light-dimmed, low-noise laboratory (located at the Kiel University Hospital) and performed with Presentation^®^ software (Version 22.0, Neurobehavioral Systems, Inc., Berkeley, CA, www.neurobs.com).

### Ratings of the visual stimuli

At the end of the study, we showed all participants 16 standardized grayscaled images from the same image set (12 images from the EEG study and 4 images from the TD task) of infant and adult animals and humans (two per species and target). Subjects were asked to rate the images using a modified version of the Self-Assessment Manikin (SAM^71^) for sexual arousal, valence, and unspecific arousal on a nine-point Likert-type scale. This procedure was adapted from Ponseti et al.^7^.

### TD data analysis

All statistical analyses were conducted with IBM SPSS Statistics 28.0 (IBM Corp. Released 2021. Armonk, NY: IBM Corp)). All data were tested for a significant deviation from normal distribution with the Shapiro-Wilk-Test. First, the error rates of response times in all conditions were calculated. There were increased error rates above 50 % of all replicates (25 per condition; cut-off: 12.5) in the animal conditions across all subjects. Pedohebephilic and teleiophilic participants did not differ significantly in the error rates (all *p *> 0.12). The mean values of the correct trials are shown in Table S4. Since both age conditions (adult vs. infant) per species would have to be at least above 50 % correct responses, all animal conditions were excluded from the analysis and only the results for the analysis of the human stimuli are reported.

One subject was excluded from the analysis since the mean progesterone concentration was identified as an outlier about the overall mean value (*M*_*subject *_= 267.10 pg/mL, *M*_overall _= 41.64 pg/mL [*SD *= 21.48]). Another subject showed ambiguous sexual age preference according to self-report, VT profile, and his young age. Originally, he was assigned to the pedohebephilic group due to his self-reported, predominantly hebephilic tendencies. However, after reassessment of the subject’s group membership by an experienced sex therapist from the “*Kein Täter werden*”-project, the subject was subsequently assigned to the teleiophilic group, primarily due to his declining VT times in Tanner stages 4–5. Response times for the human targets were analyzed with a 2 (*group affiliation*: pedohebephile vs. teleiophile) × 2 (*target*: adult vs. infantile) repeated measure ANOVA with the respective hormone concentration (testosterone, cortisol, progesterone, and estradiol) as covariates. For transparency, the results of the ANOVA with both group assignments of the respective subject are presented in Table S2 (see supplementary material). All other results are reported with the final group assignment of *N *= 42 subjects (20 pedohebephilic and 22 teleiophilic subjects). Since the effect of facial features on the attention reaction towards faces of different age groups may be modulated by motivations, preferences, and prior experience, e.g., relationship with a child or age group^76^, interest in infants^20^ and pet ownership^17,77^, we exploratively controlled for the sexual orientation of the participants, pet ownership and having children. Following Holtfrerich et al.^16^, we additionally calculated relative RTs (Delta-RTs) by subtracting the low distracting condition *per definitionem* (i.e., infant target and three adult distractors) from the high distracting condition (i.e., adult target and three baby faces as distractors). The higher the relative RT the more the participant was attentionally drawn to the baby schema. We used the Greenhouse-Geisser corrected values, if the sphericity assumption was not met. Post hoc *t*-tests were used for follow-up comparisons. The mean hormone concentrations from the aliquot were correlated with RTs using Pearson correlations. Thus, we performed correlations for human targets in the adult and infant condition across all subjects and, in a second step, separately by group. Significances are reported two-tailed if not otherwise indicated and one-tailed in case of clear a priori assumptions.

### SAM data analysis

The means and standard deviations of ratings for sexual arousal, valence, and unspecific arousal were calculated separately for the pedohebephilic and teleiophilic group. Then, a 2 × 2 × 3 repeated measure ANOVA was conducted with group (pedohebephile vs. teleiophile) as a between-subject factor and target (adult human vs. infant human) and rating (sexual arousal vs. valence vs. unspecific arousal) as within-subject factors. We used the Greenhouse-Geisser corrected values, if the sphericity assumption was not met. If a significant effect was found in the ANOVA, Bonferroni-corrected *t* tests were computed to follow up on the effects.

### Supplementary Information

Supplementary Tables.

Supplementary Information 2.

Supplementary Information 3.

## Data Availability

The fully anonymized data as well as the syntax for the TD analysis are included in this published article [and its supplementary information files].
