# Supplementary material for: Modulation of the attentional response to baby schema by testosterone in pedohebephilic men and its relation to the nurturing system
Source: Sci Rep. 2024 Jul 16;14:16385. doi: 10.1038/s41598-024-65572-6 (PMC11252362; doi:10.1038/s41598-024-65572-6)
Supplement: Supplementary file 1 — Supplementary Tables. [file 41598_2024_65572_MOESM1_ESM.docx]

**Supplementary Material**

for

**Modulation of the attentional response to baby schema by testosterone in pedohebephilic men and its relation to the nurturing system**

# Ronja Zannoni^1*^, Julian Keil^2^, Jorge Ponseti^3^, Aglaja V. Stirn^4^, Sarah K.C. Holtfrerich^5**^ and Esther K. Diekhof^6**^

^1^Institute for Sexual Medicine and Forensic Psychiatry and Psychotherapy, Centre for Integrative Psychiatry, UKSH, Kiel, 24105, Germany

^2^Department of Psychology, Christian-Albrechts-University Kiel, 24118, Germany

^3^Institute for Sexual Medicine and Forensic Psychiatry and Psychotherapy, Centre for Integrative Psychiatry, UKSH, Kiel, 24105, Germany

^4^Institute for Sexual Medicine and Forensic Psychiatry and Psychotherapy, Centre for Integrative Psychiatry, UKSH, Kiel, 24105, Germany

^5^University Hamburg, Institute of Cell and Systems Biology of Animals, Neuroendocrinology and Human Biology Unit, Hamburg, 20146, Germany

^6^University Hamburg, Institute of Cell and Systems Biology of Animals, Neuroendocrinology and Human Biology Unit, Hamburg, 20146, Germany

^*^Corresponding author: Ronja Zannoni, Institute for Sexual Medicine and Forensic Psychiatry and Psychotherapy, Centre for Integrative Psychiatry, UKSH, Kiel, 24105, Germany, [stu224170@mail.uni-kiel.de](mailto:stu224170@mail.uni-kiel.de)

^**^shared senior authorship

**Supplementary Tables**

**Table S1.** Averaged ratings of human faces for sexual arousal, valence, and unspecific arousal on a nine-point Likert-scale using the Self-Assessment Manikin (SAM; Bradley et al., 1994).

|  |  | Pedohebephilia (*n* = 20), *M* (*SD*) | | | Teleiophilia (*n* = 22), *M* (*SD*) | | |
| --- | --- | --- | --- | --- | --- | --- | --- |
|  |  | Sexual | Valence | Arousal | Sexual | Valence | Arousal |
| Human | Adult | 2.00 (1.50) | 5.50 (1.03) | 2.35 (1.57) | 1.45 (1.05) | 5.48 (.98) | 3.07 (1.94) |
|  | Infant | 1.75 (1.21) | 6.18 (1.44) | 3.30 (1.74) | 1.00 (.00) | 6.34 (1.25) | 3.18 (1.88) |

**Table S2.** Results of analysis of variance with absolute RTs for the final and initial group assignment.

| Final group assignment  (*n* = 20 pedohebephilic men, *n* = 22 teleiophilic men) | | | | | | Initial group assignment  (*n =*21 pedohebephilic men, *n* =21 teleiophilic men) | | | | | |
| --- | --- | --- | --- | --- | --- | --- | --- | --- | --- | --- | --- |
| Effect | *Df* | *SS* | *F* | *p* | ηp² | | *Df* | *SS* | *F* | *p* | ηp² |
| target | 1, 40 | 43110.07 | 7.48 | .009^*^ | .157 | | 1, 40 | 46671.17 | 7.68 | .008^*^ | .161 |
| target × group | 1, 40 | 29367.18 | 5.09 | .030^*^ | .113 | | 1, 40 | 16966.49 | 2.79 | .103 | .065 |

^*^*p* < .05 (two-sided).

**Table S3.** Demographic, sexual, clinical, and legal information about the participants.

|  | Pedohebephilia  (*n* = 20*)* | Teleiophilia  (*n* = 23) | *t* test |
| --- | --- | --- | --- |
| Age [years], *M* (*SD*) | 40.30 (15.60) | 34.04 (9.94) | *t*(31.40) = 1.54, *p* = .133,  *d* = .486^a^ |
| IQ, *M* (*SD*) | 109.45 (17.91) | 115.83 (17.88) | *t*(41) = -1.17, *p* = .251,  *d* = -.356 |
| Height [cm], *M* (*SD*) | 183.60 (6.64) | 182.09 (4.79) | *t*(41) = .87, *p* = .392,  *d* = .264 |
| EHI Laterality Index, *M* (*SD*) | 66.78 (51.53) | 86.15 (16.50) | *t*(22.38) = -1.61, *p* = .121,  *d* = -.522^a^ |
| Hetero-/homo-/bisexual, *n* (%) | 11 (55)/5 (25) /4 (20) | 20 (87)/1 (4.3)/2 (8.7) |  |
| Exclusive/non-exclusive, *n* (%) | 3 (15)/17 (85) | 18 (78.26)/5 (21.74) |  |
|  | Pedohebephilia | Teleiophilia |  |
| (Additional) paraphilias, *n* (%) |  |  |  |
| Exhibitionism | 1 (5) | 0 |  |
| Fetishism | 5 (25) | 3 (13) |  |
| Frotteurism | 2 (10) | 0 |  |
| Masochism | 0 | 0 |  |
| Sadism | 3 (15) | 2 (8.7) |  |
| Transvestism | 0 | 2 (8.7) |  |
| Sexual assault | 3 (15) | 0 |  |
| Zoophilia | 3 (15) | 0 |  |
|  | Pedohebephila | Teleiophilia |  |
| Comorbidities, *n (%)* |  |  |  |
| Major Depressive Syndrome | 7 (35) | 0 |  |
| Somatoform Disorder | 0 | 0 |  |
| Other Depressive Syndrome | 3 (15) | 0 |  |
| Panic Syndrome | 4 (20) | 0 |  |
| Other Anxiety Syndrome | 3 (15) | 0 |  |
| Bulimia Nervosa | 0 | 0 |  |
| Binge Eating Disorder | 2 (10) | 1 (4.3) |  |
| Alcohol Abuse | 4 (20) | 0 |  |
| Delinquencies, *n* (%) |  |  |  |
| Sexual offenses | 7 (33.3)^b^ | 0 |  |
| Violent offenses | 2 (9.5) | 0 |  |

^a^ Welch’s correction due to unequal variances (as indicated by Levene test, center = median)

^b^ Four subjects had prior convictions for sexual abuse of minors (up to age 15), one of whom had additional conviction for possession of child pornography. Two others were charged exclusively with possession of child pornography. Another subject had a criminal record for voyeurism of an adult victim.

**Table S4.** Mean values of the correct trials out of 25 trials per condition.

|  | Human adult | Human infant | Cat adult | Cat infant | Dog adult | Dog infant |
| --- | --- | --- | --- | --- | --- | --- |
| Total sample (*n* = 43), *M* (*SD*) | 23.21^**^ (1.54) | 22.14^**^ (1.85) | 10.19^*^  (6.28) | 9.14^**^ (5.55) | 9.89^*^ (6.57) | 13.39 (5.77) |

^**^*p* < .001; ^*^*p* < .05 (deviation from cut-off value 12.5, two-sided).
