# Supplementary material for: Modulation of the attentional response to baby schema by testosterone in pedohebephilic men and its relation to the nurturing system
Source: Sci Rep. 2024 Jul 16;14:16385. doi: 10.1038/s41598-024-65572-6 (PMC11252362; doi:10.1038/s41598-024-65572-6)
Supplement: Supplementary file 3 — Supplementary Information 3. [file 41598_2024_65572_MOESM3_ESM.pdf]

```
* Encoding: UTF-8.
GET
  FILE='C:\Users\Ronja\Desktop\RT dataset.sav'.
DATASET NAME DataSet1 WINDOW=FRONT.
DATASET ACTIVATE DataSet1.

SAVE OUTFILE='C:\Users\Ronja\Desktop\RT dataset.sav'
  /COMPRESSED.
SET TLook=None FOOTNOTE=ON AUTORECOVERY=ON Small=0.0001 SUMMARY=None
THREADS=AUTO SIGLESS=YES TFit=Both DIGITGROUPING=No LEADZERO=No.
FREQUENCIES VARIABLES=group
  /ORDER=ANALYSIS.

T-TEST GROUPS=group(1 2)
  /MISSING=ANALYSIS
  /VARIABLES=human.adult human.infant
  /ES DISPLAY(TRUE)
  /CRITERIA=CI(.95).

EXAMINE VARIABLES=human.adult human.infant BY main_sex_orientation
  /PLOT NPPLOT
  /STATISTICS DESCRIPTIVES
  /CINTERVAL 95
  /MISSING LISTWISE
  /NOTOTAL.

EXAMINE VARIABLES=human.adult human.infant BY group
  /PLOT NPPLOT
  /STATISTICS DESCRIPTIVES
  /CINTERVAL 95
  /MISSING LISTWISE
  /NOTOTAL.

GLM human.adult human.infant BY group main_sex_orientation
  /METHOD=SSTYPE(3)
  /INTERCEPT=INCLUDE
  /PRINT=DESCRIPTIVE ETASQ HOMOGENEITY
  /CRITERIA=ALPHA(.05)
  /DESIGN=group main_sex_orientation.

GLM human.adult human.infant BY group vp_kids
  /METHOD=SSTYPE(3)
  /INTERCEPT=INCLUDE
  /PRINT=DESCRIPTIVE ETASQ HOMOGENEITY
  /CRITERIA=ALPHA(.05)
  /DESIGN=group vp_kids.

GLM human.adult human.infant BY group vp_domestic_animal
  /METHOD=SSTYPE(3)
  /INTERCEPT=INCLUDE
  /PRINT=DESCRIPTIVE ETASQ HOMOGENEITY
```

```
  /CRITERIA=ALPHA(.05)
  /DESIGN=group vp_domestic_animal.

GLM human.adult human.infant BY group
  /WSFACTOR=target 2 Polynomial
  /METHOD=SSTYPE(3)
  /EMMEANS=TABLES(OVERALL)
  /EMMEANS=TABLES(group) COMPARE ADJ(BONFERRONI)
  /PRINT=DESCRIPTIVE ETASQ HOMOGENEITY
  /CRITERIA=ALPHA(.05)
  /DESIGN= group.

T-TEST
  /TESTVAL=0
  /MISSING=ANALYSIS
  /VARIABLES=human.adult human.infant
  /ES DISPLAY(TRUE)
  /CRITERIA=CI(.95).

GLM human.adult human.infant BY group WITH testosterone
  /WSFACTOR=target 2 Polynomial
  /METHOD=SSTYPE(3)
  /EMMEANS=TABLES(OVERALL) WITH(testosterone=MEAN)
  /EMMEANS=TABLES(group) WITH(testosterone=MEAN)COMPARE ADJ(BONFERRONI)
  /PRINT=DESCRIPTIVE ETASQ HOMOGENEITY
  /CRITERIA=ALPHA(.05)
  /DESIGN= group testosterone group*testosterone.

UNIANOVA delta_human_ab BY group WITH testosterone
  /METHOD=SSTYPE(3)
  /INTERCEPT=INCLUDE
  /EMMEANS=TABLES(OVERALL) WITH(testosterone=MEAN)
  /EMMEANS=TABLES(group) WITH(testosterone=MEAN) COMPARE ADJ(BONFERRONI)
  /PRINT ETASQ DESCRIPTIVE HOMOGENEITY
  /CRITERIA=ALPHA(.05)
  /DESIGN=group testosterone.

GLM human.adult human.infant BY group WITH progesterone
  /WSFACTOR=target 2 Polynomial
  /METHOD=SSTYPE(3)
  /EMMEANS=TABLES(OVERALL) WITH(progesterone=MEAN)
  /EMMEANS=TABLES(group) WITH(progesterone=MEAN)COMPARE ADJ(BONFERRONI)
  /PRINT=DESCRIPTIVE ETASQ HOMOGENEITY
  /CRITERIA=ALPHA(.05)
  /DESIGN= group progesterone.

GLM human.adult human.infant BY group WITH estradiol
  /WSFACTOR=target 2 Polynomial
  /METHOD=SSTYPE(3)
  /EMMEANS=TABLES(OVERALL) WITH(estradiol=MEAN)
  /EMMEANS=TABLES(group) WITH(estradiol=MEAN)COMPARE ADJ(BONFERRONI)
```

```
  /PRINT=DESCRIPTIVE ETASQ HOMOGENEITY
  /CRITERIA=ALPHA(.05)
  /DESIGN= group estradiol.

GLM human.adult human.infant BY group WITH cortisol_m
  /WSFACTOR=target 2 Polynomial
  /METHOD=SSTYPE(3)
  /EMMEANS=TABLES(OVERALL) WITH(cortisol_m=MEAN)
  /EMMEANS=TABLES(group) WITH(cortisol_m=MEAN)COMPARE ADJ(BONFERRONI)
  /PRINT=DESCRIPTIVE ETASQ HOMOGENEITY
  /CRITERIA=ALPHA(.05)
  /DESIGN= group cortisol_m.

GLM human.adult human.infant BY group WITH testosterone
  /WSFACTOR=target 2 Polynomial
  /METHOD=SSTYPE(3)
  /EMMEANS=TABLES(OVERALL) WITH(testosterone=MEAN)
  /EMMEANS=TABLES(group) WITH(testosterone=MEAN)COMPARE ADJ(BONFERRONI)
  /PRINT=DESCRIPTIVE ETASQ HOMOGENEITY
  /CRITERIA=ALPHA(.05)
  /DESIGN= group testosterone.

MEANS TABLES=testosterone progesterone estradiol cortisol_m
  /CELLS=MEAN COUNT STDDEV.

T-TEST GROUPS=group(1 2)
  /MISSING=ANALYSIS
  /VARIABLES=testosterone progesterone estradiol cortisol_m
  /ES DISPLAY(TRUE)
  /CRITERIA=CI(.95).

CORRELATIONS
  /VARIABLES=testosterone progesterone estradiol cortisol_m delta_human_ab
  /PRINT=TWOTAIL NOSIG FULL
  /STATISTICS DESCRIPTIVES
  /MISSING=PAIRWISE.

SORT CASES  BY group.
SPLIT FILE LAYERED BY group.

SORT CASES  BY group.
SPLIT FILE LAYERED BY group.
CORRELATIONS
  /VARIABLES=testosterone progesterone estradiol cortisol_m delta_human_ab
  /PRINT=TWOTAIL NOSIG FULL
  /STATISTICS DESCRIPTIVES
  /MISSING=PAIRWISE.

CORRELATIONS
  /VARIABLES=testosterone progesterone estradiol cortisol_m delta_human_ab
  /PRINT=ONETAIL NOSIG FULL
```

```
  /STATISTICS DESCRIPTIVES
  /MISSING=PAIRWISE.

CORRELATIONS
  /VARIABLES=testosterone progesterone estradiol cortisol_m human.adult human.infant
  /PRINT=TWOTAIL NOSIG FULL
  /STATISTICS DESCRIPTIVES
  /MISSING=PAIRWISE.

SPLIT FILE OFF.
CORRELATIONS
  /VARIABLES=testosterone progesterone estradiol cortisol_m human.adult human.infant
  /PRINT=TWOTAIL NOSIG FULL
  /STATISTICS DESCRIPTIVES
  /MISSING=PAIRWISE.

UNIANOVA delta_human_ab BY group WITH testosterone
  /METHOD=SSTYPE(3)
  /INTERCEPT=INCLUDE
  /PRINT ETASQ DESCRIPTIVE HOMOGENEITY
  /CRITERIA=ALPHA(.05)
  /DESIGN=group testosterone.

UNIANOVA delta_human_ab BY group WITH progesterone
  /METHOD=SSTYPE(3)
  /INTERCEPT=INCLUDE
  /PRINT ETASQ DESCRIPTIVE HOMOGENEITY
  /CRITERIA=ALPHA(.05)
  /DESIGN=group progesterone.

UNIANOVA delta_human_ab BY group WITH estradiol
  /METHOD=SSTYPE(3)
  /INTERCEPT=INCLUDE
  /PRINT ETASQ DESCRIPTIVE HOMOGENEITY
  /CRITERIA=ALPHA(.05)
  /DESIGN=group estradiol.

UNIANOVA delta_human_ab BY group WITH cortisol_m
  /METHOD=SSTYPE(3)
  /INTERCEPT=INCLUDE
  /PRINT ETASQ DESCRIPTIVE HOMOGENEITY
  /CRITERIA=ALPHA(.05)
  /DESIGN=group cortisol_m.

T-TEST GROUPS=group(1 2)
  /MISSING=ANALYSIS
  /VARIABLES=delta_human_ab
  /ES DISPLAY(TRUE)
  /CRITERIA=CI(.95).
```
